# Dietary Patterns of Breakfast Consumption Among Chilean University Students

**DOI:** 10.3390/nu12020552

**Published:** 2020-02-20

**Authors:** Ximena Díaz-Torrente, Daiana Quintiliano-Scarpelli

**Affiliations:** Facultad de Medicina Clínica Alemana, Universidad del Desarrollo, Santiago 7610658, Chile; xvdiaz@udd.cl

**Keywords:** breakfast quality, dietary patterns, nutritional status, factor analysis

## Abstract

Breakfast is one of the most important meals of the day. A good quality breakfast must include dairy products, cereals, and fruits. The aim of this study is to determine breakfast dietary patterns and their nutritional quality among Chilean university students. A cross-sectional non-probabilistic study was conducted in 200 university students between 18 and 27 years in Santiago, Chile. To identify dietary patterns and breakfast quality, a breakfast food survey was conducted. Patterns were identified by factor analysis. Most of the subjects (53%) ate breakfast daily, with a higher prevalence among females (60.2% vs. 43.7%, *p* < 0.05); 68% did not consume fruits and 17.5% had good breakfast quality, with no differences by sex. Four breakfast dietary patterns were identified: “dairy & cereals”, “healthy”, “traditional salty” and “traditional sweet” that together explained 35.6% of the total variance. There was no sex difference in predominant dietary patterns. The “dairy & cereals” and “traditional sweet” patterns were associated with regularly eating breakfast (β: −0.47, *p* = 0.001; β: −0.32, *p* = 0.020) and the “healthy” pattern with BMI ≥25 kg/m^2^ (β: 0.35, *p* = 0.024). In conclusion, breakfast quality was inadequate due to low fruit consumption and energy intake. The four identified patterns included cereals, bread, dairy, fats and sugars. Results may be usual in the planning of future interventions aimed at improving breakfast consumption and quality in university students.

## 1. Introduction

The transition from secondary school to a university environment involves a significant life change for students that may be accompanied by behaviors that generate health risks, such as changes in sleep patterns, reduced physical activity, increased alcohol and cigarette consumption, and poor eating habits [1,2,3,4]. Thus, this is a period of great nutritional vulnerability that is critical for the development of eating habits [5,6]. Inadequate eating habits are considered a major risk factor for obesity onset and other chronic noncommunicable diseases, with serious repercussions during adulthood [7,8].

Breakfast is defined as the first meal of the day. It is the meal that is eaten before or at the start of daily activities, within 2 hours of waking, and usually no later than 10 a.m. [9]. Breakfast is considered a good source of energy and nutrients that should provide between 20% and 25% of daily energy needs and a balanced proportion of carbohydrates, proteins, and lipids [10,11,12]. In order to allow adequate physical and intellectual performance, breakfast should be consumed in the early hours of the morning and be in accordance with the customs and habits of the population in question [13,14]. It has been shown that breakfast skipping may be linked to the up-regulation of appetite later in the day, poorer overall dietary quality [15], and altered expression of genes related to circadian rhythm, which result in an increased postprandial glycemic response [16]. In addition, food consumption between meals increases, with evidence of higher consumption of foods of low nutritional value [17]. Moreover, increased risk of obesity and related health conditions have been associated with breakfast skipping and late-night eating [18,19,20], indicating morning energy intake may have substantial health benefits [21].

To be considered good quality, breakfast should contain at least three of the main food groups: dairy, cereals, and fruits [22]. Despite the importance of breakfast, skipping breakfast or consuming a low-quality breakfast is a common practice among students [5,23,24]. Data from Chile shows that among young (19–29 years) Chileans, breakfast consists mainly of fruits (54.4%) and cereals (40.0%), with low consumption of dairy products (23.0%) [25]. This eating behavior could impact the nutritional status of the students. Weight gain during the first 1.5 years of the university period has been recognized, which can range between 1.9 and 4.2 kg [26]. Vadeboncoeur et al. [27] showed that 60.9% of students experience a weight gain of 3.4 kg (CI = 2.9–3.9 kg) during the first five years of university life.

Traditional nutritional analyses consider the consumption of specific nutrient or food groups to determine the dietary habits of the population. In recent years, determining food patterns when analyzing the human diet has gained relevance as it considers the correlation of all foods at the same time. In addition, dietary patterns are more predictive of disease risks and help build dietary guidelines [28]. The food patterns of Chilean university students have not been previously described. Thus, the objective of this study was to identify breakfast food patterns and characterize breakfast quality among Chilean university students.

## 2. Materials and Methods 

A descriptive, observational, cross-sectional study was conducted in a non-probabilistic sample of 200 university students (113 women and 87 men) of a private university of the Metropolitan Region of Santiago, Chile, from August to October 2017. The exclusion criterion was that the subjects presented some pathological condition and/or lifestyle that conditioned their eating habits, or, for females, being pregnant. Students were invited to participate via posters displayed throughout the campus and via university social networks. Interested students were encouraged to approach a stand where data collection took place. There, trained research personnel explained the study details. No student that approached the data collection stand refused participation. All subjects provided written informed consent for inclusion before they participated in the study. No participant dropped out of the study after informed consent. The study was conducted in accordance with the Declaration of Helsinki and the protocol was approved by the Ethics Committee of the Universidad del Desarrollo, Facultad de Medicina Clínica Alemana (PG_68-2017).

Anthropometry was objectively measured. Participants were weighed in light clothing and bare feet using a SECA^®^813 digital scale (Hamburg, Germany) with 100 g precision. Height was measured in the Frankfurt position with a 1-mm precision SECA^®^213 height rod (Hamburg, Germany). Body Mass Index (BMI) was calculated using the formula weight (kg)/height (m)^2^, and the value was classified according to the ranges established by the World Health Organization (WHO) to define nutritional status [29].

Participants completed a validated food survey [30] composed of two sections: subject characterization (age, sex, neighborhood residence, and major), and breakfast quality and quantity. The Photographic Atlas of Chilean Food and Preparations was used to quantify food portions reported in the survey [31]. Regularly eating breakfast was defined as having breakfast between 5 and 7 times a week. Classification of breakfast quality was performed according to EndKid Study criteria [22], which considers that a good breakfast should be composed of dairy products, cereals and fruit. A “regular” breakfast quality includes two of the mentioned groups, an “insufficient” quality includes only one group and a “poor” is when breakfast is not consumed or not includes these foods groups [32]. To determine the nutritional composition of breakfast, caloric and macronutrient intake was calculated according to food exchanges [33].

To determine the percentage of the ideal energy intake that the breakfast calories and macronutrients represent, energy expenditure was calculated using the FAO/WHO/UN formula [34], considering ideal weight for those subjects who did not have a normal nutritional status and real weight for normal-weight subjects (body mass index 18.5–24.9 kg/m^2^). The formula includes a physical activity level, which was not measured as part of the current study. Considering that recent data have shown that over 80% of the Chilean population aged 20–29 years are sedentary [35], for the purposes of the formula, we applied the sedentary level (1.4) of physical activity.

The socioeconomic level was categorized as high, medium, or low by neighborhood of residence, according to the socioeconomic map of Santiago. The map is based on public information provided by population-level surveys [36].

### Statistical Analysis

A descriptive analysis of each variable was performed according to variable type (qualitative, by relative and absolute frequency and quantitative by measures of central tendency and dispersion). The normality of variables was analyzed by the Shapiro–Wilk test. For bivariate analyses, chi-square, Fisher’s Exact (when <5 observations) and Mann–Whitney U tests were used. The identification of food patterns was carried out through principal component factor analysis. The adequacy of the sample was verified by the Kaiser–Meyer–Olklin test (KMO) and applicability of factor analysis was evaluated by Bartlett’s sphericity test. The number of patterns was extracted by Kaiser criterion, sedimentation graph, and composition interpretation. Varimax orthogonal rotation was performed to achieve a simpler structure, facilitating the interpretation of the data. Factor loadings greater than 0.30 were retained. The greater the magnitude of the loading, the greater the contribution in the pattern; negative factor loadings were considered inversely associated with the pattern [37]. Scores were derived for each factor; the highest value indicated the predominant pattern for each subject.

Multiple linear regression analyses were performed to determine significant associations among the identified factor dietary patterns scores. First, univariate analysis tested the association between each food pattern with sex, breakfast frequency (regularly or not), and nutritional status (BMI <25 kg/m^2^ or BMI ≥25 kg/m^2^) as independent variables. Independent variables with *p*-values ≤0.20 were selected for subsequent multiple regression models using a stepwise forward procedure. Final regression models included only variables with *p*-values <0.05, with the exception of socioeconomic level and age, which were included in models independent of *p*-value. In the four final models, the homoscedasticity was confirmed by the Breusch–Pagan/Cook–Weisberg test. The level of statistical significance that was considered for all analyses was *p* < 0.05. Stata 14.1 software (Texas, USA) was used for analyses.

## 3. Results

The sample consisted of 200 students, 57% female with an average age of 21.1 (±1.9) years. The general characteristics of the sample are presented in Table 1. In relation to the nutritional status of university students, 71.7% of the women and 56.3% of the men were normal weight (*p* = 0.024). The majority of participants were of a high socioeconomic level (74.5%). Most participants were from the areas of humanities (32%), health (21.5%) and engineering (21.5%). There was a significant difference by sex, with more women than men studying in the area of humanities (38% vs. 24.1%) and more men than women in engineering (34.4% vs. 11.5%, *p* < 0.001). Regarding the frequency of breakfast, 53% of the sample regularly had breakfast (5–7 times per week), with a statistically significant difference by sex (*p* = 0.049). The foods that defined breakfast quality—dairy, cereals, and fruits—were present in 66.5%, 76%, and 31.5% of the sample, respectively. We observed a significant sex difference for the consumption of fruit at breakfast, with 37.2% of women reporting eating fruit at breakfast versus 24.1% of men (*p* = 0.049). Only 17.5% of the subjects had a good quality breakfast, with no difference by sex.

The nutritional composition of breakfast is shown in Table 2. Median energy intake was 297.6 kcal (211.5) for women and 368 kcal (246.5) for men, with no statistically significant difference. These calories represent 15.4% and 14.2%, respectively, of participant total energy requirements. The nutrients in breakfast consumption that presented a significant difference by sex were grams of proteins (*p* = 0.014), with women consuming 11.5 g (13.5) compared to 15.4 g (17.0) in men; and grams carbohydrates (*p* = 0.038) 45.8 g (34.8) and 56.8 g (39.2) for women and men, respectively. Macronutrient percentage was not statistically different by sex.

Table 3 shows the four breakfast patterns retained for the sample, named according to their food combination. Pattern 1, “dairy & cereals”, which explained 9.9% of the total variance, was characterized by consumption of yogurt or milk and breakfast cereals. Pattern 2, “healthy”, explained 9.5% of the total variance and consisted of sliced bread, ham, avocado and low-fat white cheese. Pattern 3, “traditional salty”, explained 8.2% of the total variance and contained traditional Chilean white bread (*marraqueta*/*hallulla*), ham, cheese and sugar. Pattern 4, “traditional sweet”, explained 8.0% of the total variance, included traditional white bread, butter and jam.

Results of the linear regression models for individual characteristics (sex, breakfast frequency, and nutritional status) related to food factor scores are reported in Table 4. Higher factor scores related to more frequent consumption of the included foods in each pattern. Eating breakfast regularly was significantly associated with “dairy & cereals” and “traditional sweet” food patterns (univariate and multivariate models). Overweight status was associated with the “healthy” food pattern. Sex did not relate to any food patterns.

## 4. Discussion

The purpose of the study is to determine breakfast food patterns and characterize breakfast quality among Chilean university students. We have identified 4 distinct breakfast patterns: “dairy & cereals”, “healthy”, “traditional salty” and “traditional sweet”. To our knowledge, this is the first study to provide information on food patterns in this population group. Our main results showed that eating breakfast regularly was related to “dairy & cereals” and “traditional-sweet” patterns, while being overweight was related to the “healthy” food pattern. In relation to breakfast quality, participants reported low fruit and energy intake.

The number of university students in Chile has doubled in the last decade, representing a significant proportion of the youth population [38]. Dietary habits at this life stage are decisive for quality of life and future health [39,40]. Breakfast is known for being one of the meals most omitted by young people [41]. A 43% higher risk of obesity has been observed in children and adolescents who skip breakfast versus those who eat breakfast regularly [42]. On the other hand, several benefits have been observed in adolescents in relation to regular breakfast consumption, for example, lower body fat [43], higher grip strength, and faster sprint times [44]. In addition, good breakfast quality is related to greater academic [45] and physical performance [46].

In our study, we determined breakfast consumption patterns among university students using factor analysis. This type of analysis integrates multivariate statistical methods. We used information reported in food records to identify common patterns of food consumption among participants. The breakfast food patterns we identified explained 37.5% of the variance, which, although is below the recommended levels (60%) [47], is to be expected as the human diet presents a great variety of food or food groups. In addition, the literature shows that the proportion of variance explained varies depending on sample size. For example, Maia et al. [48] studied 10,926 adolescents and, using the same methods, identified two consumption patterns that explain 50% of the variance; despite this limitation, our study is the first of this type in Chilean university students.

We did not observe a statistically significant sex difference in breakfast patterns but did observe a predominant consumption for the "traditional salty" pattern, composed mainly of high energy density foods, in men. Similar findings have been shown among Australian students who have a high intake of nutrient-poor and high-energy foods [49]. This suggests that university men make poorer food choices.

On the other hand, the “dairy & cereals” and “traditional sweet” patterns were positively associated with regularly breakfast consumption, which may be because these foods are easily accessible at breakfast time or that they require less time to prepare, considering that the time available to eat breakfast is one of the main barriers of not doing so [50]. Dairy products, cereals, and sweets (sugars) were also common breakfast foods reported by Spanish university students [51]. The results of Del Piero et al. [52] showed that the diet of Argentine university students moves away from a pattern of healthy eating since it is based primarily on processed, industrialized, and micronutrient-poor products. Similar to what has been reported by Pastor et al. [53] in Spain, Whatnall et al. [49] in Australia, and Blondin et al. [54] in the US, patterns that include processed foods explained most of the total variance (22.2%, 16.8%, and 10.3% respectively); we found a "dairy & cereals" pattern that contained a high proportion of processed breakfast cereals. Thus, food patterns of university students may be similar, independent of the continent where they live.

The “healthy” pattern included foods such as avocado, which has been associated with beneficial effects on human health at the cardiometabolic level and has demonstrated antioxidant effects [55], and low fat white cheese (“*quesillo*”), a dairy derivative with low saturated fat content. Both foods represent a positive dietary choice. Surprisingly, the “healthy” pattern was associated with a BMI of ≥25 kg/m^2^. Possible explanations for this result are that overweight subjects could have misreported their breakfast consumption, potentially reporting more socially desirable (health) consumption [56]. It is also possible that these subjects were attempting to improve their health. A prospective study design could further elucidate these findings.

When defining the quality of breakfast for our sample of Chilean university students, we found that quality was lacking, mainly due to the lack of fruit. Only 35.5% of subjects incorporated fruit at breakfast and those that did were mainly women. Crovetto et al. [57] showed that Chilean university students have low fruit–vegetable consumption when compared to the food guidelines for this population. Olivares et al. [58] identified some of the barriers posed by university students to not incorporating fruits into their diet: “I live alone and I feel lazy to prepare them”, “I don’t have time” and “I forget to eat them”. These barriers highlight the need for dietary strategies and time organization skills aimed at resolving these barriers. The case study conducted by Anigstein [59] in Chilean university students indicates that students have a diet low in fruits, vegetables, and fiber, which varies depending on personal situation, the context in which eating takes place, and food availability. Sánchez et al. [60] suggest that educational sessions are able to improve nutrition knowledge among university students. Thus, reinforcing interventions aimed at complying with food guidelines in relation to fruit consumption should be considered in order to improve meal quality among these subjects.

Regarding the frequency of daily breakfast consumption, an important proportion of our sample did not eat breakfast every day (53%). It has been reported that the daily frequency of breakfast consumption is associated with healthy lifestyles. Examples include the work of Corder et al. [61] that indicates that adolescents who consume breakfast daily have a higher level of physical activity compared to those who do not consume. A study by Keski-Rakonen et al. [62] observes that adolescents and adults who do not consume breakfast have a lower level of physical activity. This suggests that breakfast may be a promoter of physical activity. Although, in our study, we did not incorporate physical activity, in a future study, it would be interesting to see how the habit of eating breakfast may promote healthy life behaviors among Chilean university students.

Studies have suggested that subjects who do not usually eat breakfast may have decreased fullness, decreased levels of satiety hormones [63], and consume a higher amount of calories in the afternoon/evening hours [64]. In a study using data from NHANES 2005–2010, Kant and Graubard [65] report that the difference in calories in a 24-hour period, comparing a day with breakfast and a day without, was 247 and 187 kcal, respectively, and that energy intake at lunch was higher on the day without breakfast (202 kcal in men and 121 kcal in women), showing a caloric compensation behavior at lunchtime. Using a randomized controlled trial design, Nas et al. [66] showed that subjects who do not have breakfast have higher postprandial insulin concentrations after lunch and an increase in fat oxidation during the day compared to subjects who do not consume dinner, which suggests the development of a metabolic inflexibility in response to a prolonged fast that, in the long term, can lead to low-grade inflammation and an altered glucose homeostasis. These studies show compensatory eating behavior at lunchtime in those subjects who do not consume breakfast, which may lead to metabolic risks.

The majority of our sample had a normal BMI. Similar results for Chilean university students have been reported in the studies of Rodríguez et al. [67] and Espinoza et al. [68], who demonstrated that 63.7% and 65% of the students, respectively, had normal a BMI. In university students in Galicia, Spain, the average BMI was 22.17 ± 3.72 kg/m^2^, with a higher BMI in men (24.16 ± 2.86 kg/m^2^) compared to women (21.35 ± 3.75 kg/m^2^) [69]. Similar data have been reported in US university students where most, especially women, were within the healthy range for bodyweight [70]. These data suggest that changes in eating habits experienced by subjects during their university period may impact BMI in later years. Our findings are consistent with data reported in the 2016–2017 Chilean National Health Survey [35], which shows a higher prevalence of overweight and obesity in age groups after the university stage. This reinforces the need for food or nutritional interventions in this group to prevent future overweight and obesity.

Calorie intake was not statistically different between men and women. Calories represented 15.4% and 14.2%, respectively, of the total caloric requirement for the subjects, showing values below those recommended for this meal. Concha et al. [71] also observed energy intakes below recommendations among university students in Valparaíso, Chile, and showed significant differences according to the body composition of participants. Specifically, participants with low and normal fat percentages, consumed close to 19% of total caloric requirement at breakfast, while participants with excess fat consumed only 8.9% of daily calories at breakfast. These findings imply that a caloric distribution of breakfast close to recommended values may be associated with health benefits, in this case, better nutritional status, and lower cardiovascular risk. In examining macronutrients, consumption of proteins and carbohydrates significantly differed by sex: 11.5 g in women and 15.4 g in men, and 45.8 and 56.8 g, respectively. These amounts are similar to those reported for Spanish university students [51]. The main protein source at breakfast was ham and dairy products. Carbohydrates included different types of bread (“*marraqueta*”/“*hallulla*”/sliced) and cereals. In the case of fats, avocado was the most commonly consumed food, which refers to the Chilean food culture. Chile is an avocado-producing country with high consumption of this food. In the case of sugars, the most consumed food was jam. Finally, protein represented 17% of calories consumed at breakfast, fats were 19%, and carbohydrates were 63%, which indicates a poor caloric distribution. The excessive intake of processed carbohydrates (bread or cereals) may be an additional point of action to promote better quality breakfast consumption.

Our results should be interpreted by taking into account the following limitations. First, we conducted the study in a private university of the Metropolitan Region of Santiago de Chile; thus the results cannot be extrapolated to all university students in the country. Second, fruits and vegetables consumed could have been influenced by seasonality, as the study was conducted at the end of the winter season in which variety is limited in our country. Third, we focused only on habitual breakfast and did not determine food consumption for the whole day. Further, in order to obtain a more precise calculation of energy expenditure, future studies may benefit from measuring, either objectively or by a validated questionnaire, physical activity. Finally, although we relied on well-established methodology, factor analysis involves subjective decision-making (e.g., the foods included, number of patterns retained, pattern labeling), which could have affected our results and their interpretation. We weighed eigenvalues, factor loadings, and explained variance more heavily in deciding how many and which factors to retain for this analysis.

## 5. Conclusions

This study provides unique data regarding the breakfast food patterns of Chilean university students. Four patterns were retained: “dairy & cereals”, “healthy”, “traditional salty” and “traditional sweet” that together explained 35.6% of the total variance. In particular, two patterns were associated with frequent breakfast consumption and one with excessive weight. Future studies that use longitudinal methods to identify changes in food patterns over the course of university life are suggested. A low proportion of students were categorized as having a good breakfast quality, mainly due to the lack of fruit consumed. Thus, the consumption of fruit as part of a quality breakfast should be subject to evaluation and promotion to improve the overall diet. These findings are relevant for future health promotion interventions and behavior change in this population.

## Figures and Tables

**Table 1:** General sample characteristics.

Variables	Overall% (*n*)	Women% (*n*)	Men% (*n*)	*p*-Value
***Nutritional status***				
Underweight	3.5 (7)	4.4 (5)	2.3 (2)	**0.024 ^1^**
Normal weight	65.0 (130)	71.7 (81)	56.3 (49)	
Overweight	31.5 (63)	23.9 (27)	41.4 (36)	
***Socioeconomic level***				
High	74.5 (149)	76.1 (86)	72.4 (63)	0.559 ^2^
Medium	19.5 (39)	19.4 (22)	19.5 (17)	
Low	6.0 (12)	4.4 (5)	8.0 (7)	
***School major***				
Arts	11.5 (23)	13.7 (15)	9.2 (8)	**<0.001 ^2^**
Communications	13.5 (27)	7.9 (9)	20.6 (18)	
Humanities	32.0 (64)	38.0 (43)	24.1 (21)	
Health	21.5 (43)	29.2 (33)	11.4 (10)	
Engineering	21.5 (43)	11.5 (13)	34.4 (30)	
***Breakfast frequency***				
Regularly (5–7 t/w)	53.0 (106)	60.2 (68)	43.7 (38)	**0.049 ^2^**
Sometimes (1–4 t/w)	41.5 (83)	36.3 (41)	48.3 (42)	
Never	5.5 (11)	3.5 (4)	8.0 (7)	
***Reason for not having breakfast***				
Not enough time	46.8 (44)	55.6 (25)	38.8 (19)	0.537 ^1^
Not hungry	21.3 (20)	15.6 (7)	26.5 (13)	
It makes me feel sick	4.3 (4)	4.4 (2)	4.1 (2)	
More than one reason	20.2 (19)	17.8 (8)	22.5 (11)	
Another reason	7.5 (7)	6.7 (3)	8.2 (4)	
***Presence of breakfast food groups***				
Dairy	66.5 (133)	68.1 (77)	64.4 (56)	0.575 ^2^
Cereals	76.0 (152)	78.7 (89)	72.4 (63)	0.297 ^2^
Fruits	31.5 (63)	37.2 (42)	24.1 (21)	**0.049 ^2^**
***Breakfast quality***				
Good	17.5 (35)	19.5 (22)	14.9 (13)	0.522 ^2^
Regular	49.5 (99)	50.4.1 (57)	48.3 (42)	
Insufficient	17.5 (35)	17.7(20)	17.2 (15)	
Poor	15.5 (31)	12.4 (14)	19.5 (17)	

^1^ Fisher exact test; ^2^ chi-square test; t/w: times a week; bold indicates significant differences.

**Table 2:** Nutritional composition of university students’ breakfasts according to sex.

Nutrients	Overall Median (IQR)	Women Median(IQR)	Men Median (IQR)	*p*-Value
**Energy**				
kcal/day	307.0 (228.1)	297.6 (211.5)	368.0 (246.5)	0.059
%/day	14.7 (10.8)	15.4 (11.7)	14.2 (10.8)	0.110
**Protein**				
g/day	13.0 (14.8)	11.5 (13.5)	15.4 (17.0)	**0.014**
%/day	2.2 (2.7)	2.3 (3.1)	2.1 (2.4)	0.721
**Carbohydrate**				
g/day	48.4 (38.8)	45.8 (34.8)	56.8 (39.2)	**0.038**
%/day	2.2 (2.7)	2.3 (3.1)	2.1 (2.4)	0.721
**Lipid**				
g/day	6.5 (8.0)	6.5 (6.8)	7. 3 (10.0)	0.316
%/day	2.7 (3.3)	2.8 (3.5)	2.5 (3.2)	0.287

IQR: interquartile range; Mann–Whitney U test; g/day: grams per day; %/day: percentage per day; bold indicates significant differences.

**Table 3:** Factor loadings for breakfast patterns according to consumed food reported by Chilean university students.

Foods	Food Patterns			
	Dairy & Cereal	Healthy	Traditional Salty	Traditional Sweet
**Dairy**				
Milk	0.773	0.045	0.045	0.157
Yogurt	0.792	−0.001	0.005	0.007
Cheese	−0.019	0.167	0.712	0.054
Low fat white cheese	−0.044	0.302	−0.336	0.069
**Cereals**				
Traditional white bread	−0.184	−0.212	0.354	0.629
Sliced bread	−0.039	0.803	0.069	−0.148
Breakfast cereals	0.704	−0.165	−0.107	−0.223
**Protein**				
Ham	−0.053	0.634	0.495	0.069
**Fat**				
Avocado	0.014	0.604	−0.282	0.192
Butter	0.030	0.231	0.073	0.668
**Fruits**				
Apple	0.090	0.028	−0.190	−0.067
Banana	0.138	−0.153	−0.060	−0.014
Orange	−0.074	0.258	−0.447	0.074
**Liquids**				
Tea	−0.238	−0.119	0.157	0.051
Coffee	−0.116	0.154	−0.012	−0.014
Juice	−0.030	−0.021	−0.049	−0.001
**Sugary products**				
Sugar	0.017	0.052	0.392	0.035
Jam	0.102	−0.087	−0.189	0.714
Pastries	0.143	−0.069	−0.089	−0.103
Explained variance (%)	9.9	9.5	8.2	8.0
% Accumulative	9.9	19.5	27.7	35.7
Eigenvalue	1.9	1.8	1.6	1.5

Factor loadings ≥0.30 were retained; Kaiser–Meyer–Olkin test = 0.51.

**Table 4:** Associations among dietary patterns scores and characteristics of Chilean university students.

Variables	Dairy & Cereals		Healthy		Traditional Salty		Traditional Sweet	
	Univariate	Multivariate ^1^	Univariate	Multivariate ^1^	Univariate	Multivariate ^1^	Univariate	Multivariate ^1^
	β (*p*)	β (*p*)	β (*p*)	β (*p*)	β (*p*)	β (*p*)	β (*p*)	β (*p*)
**Sex**								
Female	1.00	-	1.00	-	1.00	-	1.00	-
Male	0.15 (0.298)		−0.00 (0.996)		0.21 (0.146)		0.05 (0.701)	
**Breakfast frequency**								
Regularly	1.00	−0.47 (0.001)	1.00	-	1.00	-	1.00	−0.32 (0.020)
Not regularly	−0.46 (0.001)		-0.16 (0.267)		−0.08 (0.571)		−0.32 (0.023)	
**Nutritional status**								
BMI <25 kg/m^2^	1.00	-	1.00	0.35 (0.024)	1.00	-	1.00	-
BMI ≥ 25 kg/m^2^	−0.07 (0.628)		0.31 (0.039)		0.18 (0.245)		0.03 (0.831)	

^1^ Linear regression model adjusted for age and socioeconomic level; BMI: body mass index.
